# Prescription profile of potentially aristolochic acid containing Chinese herbal products: an analysis of National Health Insurance data in Taiwan between 1997 and 2003

**DOI:** 10.1186/1749-8546-3-13

**Published:** 2008-10-23

**Authors:** Shu-Ching Hsieh, I-Hsin Lin, Wei-Lum Tseng, Chang-Hsing Lee, Jung-Der Wang

**Affiliations:** 1 Division of Health Technology Assessment, Center for Drug Evaluation, Taiwan; 2 Institute of Occupational Medicine and Industrial Hygiene, College of Public Health, National Taiwan University, Taipei, Taiwan; 3 Committee on Chinese Medicine and Pharmacy, Department of Health, Executive Yuan, Taipei, Taiwan; 4 Emergency Department of Taipei City Hospital, Zhongxiao Branch, Taipei, Taiwan; 5 Department of Occupational Medicine, Ton Yen General Hospital, Hsinchu, Taiwan; 6 Department of Internal Medicine and the Department of Environmental and Occupational Medicine, National Taiwan University Hospital, Taipei, Taiwan

## Abstract

**Background:**

Some Chinese herbal products (CHPs) may contain aristolochic acid (AA) or may be adulterated by the herbs suspected of containing AA which is nephrotoxic and carcinogenic. This study aims to identify the risk and the prescription profile of AA-containing CHPs (AA-CHPs) in Taiwan.

**Methods:**

A longitudinal analysis was conducted on a randomly sampled cohort of 200,000 patients using the data from the National Health Insurance (NHI) in Taiwan between 1997 and 2003.

**Results:**

During the 7-year study period, 78,644 patients were prescribed with AA-CHPs; most patients were females, or middle-aged, or both. A total of 526,867 prescriptions were made to use 1,218 licensed AA-CHPs. Over 85% of the AA-exposed patients took less than 60 g of AA-herbs; however, about 7% were exposed to a cumulated dose of over 100 g of *Radix et Rhizoma Asari *(*Xixin*), *Caulis Akebiae *(*Mutong*) or *Fructus Aristolochiae (Madouling)*. Patients of respiratory and musculoskeletal diseases received most of the AA-CHP prescriptions. The most frequently prescribed AA-CHPs *Shujing Huoxie Tang*, *Chuanqiong Chadiao San *and *Longdan Xiegan Tang*, containing *Radix Stephaniae Tetrandrae*, *Radix et Rhizoma Asari *and *Caulis Akebiae, *respectively.

**Conclusion:**

About one-third of people in Taiwan have been prescribed with AA-CHPs between 1997 and 2003. Although the cumulated doses were not large, further actions should be carried out to ensure the safe use of AA-CHPs.

## Background

Considerable attention to the safe use of Chinese herbal medicines has been drawn since the reports of nephropathy due to some Chinese herbs [1,2]. The reported nephrotoxicity and carcinogenicity of aristolochic acid (AA) was subsequently corroborated by clinical reports [3-9], results from animal models [10-12] and the detection of AA bound DNA adducts in kidney and ureteral tissues [13-16]. These reports led to the prohibition of all AA-containing products in many countries and regions, such as the USA, UK, Canada, Germany, Australia and Taiwan [13,17-20]. The Bureau of Food and Drug Analysis in Taiwan is mandated to regularly monitor AA-containing Chinese herbal products (AA-CHPs) in the market by quantitative and qualitative analysis.

Substitution of specific AA-containing herbs has been reported. *Caulis Akebiae *(*Mutong*), *Radix Stephaniae Tetrandrae *(*Fangji*) and *Radix Aucklandiae *(*Muxiang*) may potentially be substituted by *Caulis Aristolochiae Manshuriensis *(*Guanmutong*) [21], *Radix Aristolochiae Fangchi *(*Guanfangji*) [22-24] and *Radix Aristolochiae *(*Qingmuxiang*) respectively. Inappropriate uses were reported after the ban had been imposed [18,25-28]. Containing trace amounts of AA [29,30], *Radix et Rhizoma Asari *(*Xixin*) is banned [19,31] but still available in Mainland China, Taiwan, Japan and Korea [32].

The CHPs currently covered by the National Health Insurance (NHI) of Taiwan do not include raw herbs. Manufactured and marketed as extract products, CHPs are equivalent to the 'finished herbal products' or 'mixed herbal products' as defined by the World Health Organization (WHO) [33]. In terms of safety, AA-CHPs may be quite different from individual AA herbs because traditional Chinese medicine formulae that are used to make AA-CHPs were designed to not only enhance the efficacy of the herbs but also reduce their toxicity [34,35].

This study aims to determine the prescription profile of AA-CHPs in Taiwan based on data for the period between January 1997 and November 2003. The prescription data for 2004 enable us to determine whether the ban on the use of AA herbs was complied with in Taiwan [36] where the high incidence and prevalence rates of chronic kidney disease were associated with the use of herbal medicines [37].

## Methods

### Selection of herbs

AA-CHPs in this study are defined as the Chinese herbal products that are (1) either suspected of containing AAs (AA herbs), e.g. *Herba Aristolochiae *(*Tianxianteng*), *Fructus Aristolochiae (Madouling) *and *Xixin*, or (2) likely to be adulterated by AA herbs, e.g. *Fangji*, *Muxiang *and *Mutong*. In Taiwan, the ban on some SAA herbs, including *Guanfangji*, *Qingmuxiang*, *Guanmutong*, *Madouling*, and *Tianxianteng*, took effect on 4 November 2003. However *Xixin*, *Mutong*, *Fangji *and *Muxiang*, may still be used if correct species without adulteration or malnomenclature are assured. We therefore examined all the CHPs licensed by the Committee on Chinese Medicine and Pharmacy (CCMP) between 1997 and 2003, including single herbs and herbal formulae, to determine whether they include AA herbs. The inclusion period runs from the start of the research database (1 January 1997) to one day prior to the ban on AA-CHPs (3 November 2003). The databases used in this study were also used in similar studies [38,39].

### List of licensed Chinese herbal products

The CCMP list shows that 18,019 CHPs were licensed during the study period, of which 9,837 were covered by the NHI. CHPs in Taiwan can only be prescribed by Chinese medicine practitioners and CHP prescriptions usually contain more than one single herb/herbal formula [38]. For simplicity, all CHPs with the same CCMP standard formulae are classified under the same categories, regardless of slight variations among products of different pharmaceutical companies [40]. For example, there are 46 approved licenses for the formula *Duhuo Jisheng Tang*.

### National Health Insurance reimbursement database

The NHI covers over 96.16% of the population in Taiwan [41]. Our cohort of 200,000 patients was randomly selected from all NHI beneficiaries, according to the methods of Knuth [42] and Park and Miller [43] using random numbers generated by a program written in Sun WorkShop C 5.0. Under secure encryption, all reimbursement data of the cohort from 1996 onwards were collected and analyzed. The database contains all transactions of health care services for the cohort, including both Western medicine and Chinese medicine, with the dates and some details of all outpatient visits, hospitalization, diagnoses, prescribed CHPs (dosages, dosage frequency and prescription duration) and the personal data of the patients. The database was made available by the National Health Research Institutes in 2002 and was widely used by researchers in various fields [44]. The main datasets used were 'Ambulatory care expenditure by visits', 'Details of ambulatory care orders' and 'Registry for contracted medical facilities'. As the NHI of Taiwan does not cover the use of Chinese medicine in inpatient services, we only studied the use of Chinese medicine in outpatient services. Using the data of 2004, we also studied whether Chinese medicine practitioners complied with the ban on AA herbs.

### Statistical analysis

Data analysis was undertaken by descriptive statistics, including the decomposition of the AA herb contents of the licensed and prescribed AA-CHP items, AA-CHP prescription rates stratified by patient's gender and age, the median (plus 5 and 95 percentiles) of cumulated doses of AA herbs, the population distribution of those who had been potentially exposed to AA herbs at various dosages, the frequencies of the disease categories prescribed with AA-CHPs, the most frequently prescribed herbal formulae potentially containing AA herbs, and the most common duration and dosage frequencies of AA-CHP prescriptions. All of the above analyses were performed using the SAS software package (version 9.1, USA).

## Results

Between 1 January 1997 and 3 November 2003, 1,218 (12.38%) AA-CHPs were identified out of the total of 9,837 licensed CHPs, of which the most frequently prescribed were *Muxiang *(35.3%) and *Xixin *(30.7%). A total of 526,867 cases of prescribed and reimbursed AA-CHPs were recorded (Table 1). Among all the AA-CHPs, *Xixin *was the most frequently prescribed (44.7%). The co-existence of more than two AA herbs was identified in both licensed and prescribed AA-CHPs, of which *Mutong *and *Xixin *were the most frequently seen. During the study period, 105,737 patients (52.9%) sought Chinese medicine treatment on at least one occasion, of which 78,644 were prescribed with AA-CHPs. The AA-exposed population demonstrated the prevalence of middle-aged female patients (Table 2). More than 70% of the patients were exposed to lower cumulated doses (less than 30 mg) of all AA herbs in CHPs; about 7% of the patients were prescribed with *Xixin*, *Mutong *and *Madouling *at cumulated doses of over 100 g (Table 3). Given that the random sample of this cohort accounts for approximately 1% of the population of Taiwan, it may be inferred that about 344,300 people were exposed to such high cumulated doses of *Xixin*, while about 234,700 people were exposed to similarly high cumulated doses of *Mutong*.

**Table 1:** Distribution frequencies of licensed and prescribed Chinese herbal products potentially containing aristolochic acid, 1997–2003*

	Licensed CHPs		Prescribed CHPs	
	Counts	%	Counts	%
Types of AA herbs included				
*Tianxianteng*	1	0.1	339	0.1
*Madouling*	18	1.5	1,395	0.3
*Xixin*	307	25.2	191,297	36.3
Herbs potentially adulterated by AA herbs				
*Fangji *(by *Guanfangji*)	174	14.3	93,447	17.7
*Muxiang *(by *Qingmuxiang*)	409	33.6	107,014	20.3
*Mutong *(by *Guanmutong*)	225	18.5	87,073	16.5
≥2 of above herbs				
*Mutong *and *Muxiang*	17	1.4	2,200	0.4
*Mutong *and *Xixin*	63	5.2	44,101	8.4
*Muxiang *and *Xixin*	2	0.2	-	-
*Muxiang*, *Xixin *and *Tianxianteng*	2	0.2	1	-
Total	1,218	100.0	526,867	100.0

*The table shows the distribution frequencies of licensed and prescribed Chinese herbal products (CHPs) that may potentially contain aristolochic acid (AA).

**Table 2:** Prescription frequencies of Chinese herbal products (by gender, age and types of herbs), 1997–2003*

Herbal products	Gender		Age (years)					
	Male	Female	<12	12–18	19–34	35–59	60–75	≥76
Any CHPs	35.4	41.8	11.1	7.3	23.6	26.5	7.2	1.6
AA-CHPs	25.9	31.6	6.8	5.8	17.6	20.6	5.6	1.2
Types of AA herbs included								
*Xixin*	14.8	20.2	4.9	3.1	9.8	12.7	3.7	0.8
*Madouling*	0.2	0.3	0.1	--	0.1	0.2	0.1	--
*Tianxianteng*	--	--	--	--	--	--	--	--
Herbs potentially adulterated by AA herbs								
*Fangji*	11.5	13.6	0.8	2.1	7.6	11.0	3.0	0.6
*Muxiang*	10.3	14.7	2.7	2.5	7.9	9.2	2.2	0.5
*Mutong*	10.8	14.4	3.3	2.4	7.6	9.2	2.2	0.4

*The prescription frequencies (per 1,000 person-years) of Chinese herbal products (CHPs) are stratified by gender, age and the types of AA containing herbs (AA herbs) or those potentially adulterated by AA herbs.

**Table 3:** Distribution frequencies* of Chinese herbal product prescriptions potentially containing aristolochic acid (by cumulated doses), 1997–2003

Herbal product	Cumulated dose (g) Median (90% CI^#^)	No. of patients	Percentages (%) of patients with various cumulated doses of AA herbs					
			<15 g	16–30 g	31–60 g	61–100 g	101–150 g	>150 g
Types of AA herbs included								
*Xixin*	12.6 (1.5–128.5)	47,869	54.1	18.6	13.6	6.4	3.3	4.0
*Tianxianteng*	10.5 (0.03–87.0)	110	66.0	19.4	2.9	7.8	1.9	1.9
*Madouling*	21.0 (4.0–120.0)	665	36.0	29.1	19.7	7.5	4.3	3.4
Herbs potentially adulterated by AA herbs								
*Fangji*	6.0 (1.1–50.8)	34,462	77.8	12.0	6.2	2.2	0.9	1.0
*Muxiang*	8.0 (1.2–70.0)	34,195	69.1	15.9	8.8	3.2	1.6	1.5
*Mutong*	14.0 (1.8–124.4)	34,399	51.8	19.9	14.6	6.7	3.2	3.8

*Distribution frequency refers to the number of patients who have been prescribed with Chinese herbal products that may potentially contain aristolochic acid (AA).

^#^90% CI: 90% confidence interval

The major disease categories often prescribed with AA-CHPs include respiratory diseases (132,598 visits) and musculoskeletal/connective diseases (77,153 visits), followed by symptoms/signs/ill-defined conditions (68,466 visits), digestive diseases (46,646 visits) and injury/poisoning (40,260 visits). Among all AA-CHPs, 90.7% were in the form of herbal formulae, of which the most frequently prescribed were *Shujing Huoxie Tang *(containing *Fangji*), *Chuanqiong Chadiao San *(containing *Xixin*) and *Longdan Xiegan Tang *(containing *Mutong*) (Table 4).

**Table 4:** Distribution frequencies* of the most commonly prescribed herbal formulae potentially containing aristolochic acid, 1997–2003

Herbal formula containing AA herbs	Prescription frequency		Type of AA herbs included or potentially adulterated
	Counts	%	
*Shujing Huoxie Tang*	75,472	14.3	*Fangji*
*Chuanqiong Chadiao San*	58,004	11.0	*Xixin*
*Longdan Xiegan Tang*	42,351	8.0	*Mutong*
*Xiaoqinglong Tang*	42,241	8.0	*Xixin*
*Duhuo Jisheng Tang*	41,594	7.9	*Xixin*
*Xinyi San*	39,323	7.5	*Mutong*
*Xiangsha Liujunzi Tang*	23,580	4.5	*Muxiang*
*Guipi Tang*	16,946	3.2	*Muxiang*
*Xiaofeng San*	16,186	3.1	*Mutong*
*Zhenggu Zijin Dan*	15,105	2.9	*Muxiang*
Total	526,867	100.0	

*Distribution frequency refers to the top ten most commonly prescribed herbal formulae that may potentially contain aristolochic acid.

About 97.5% of all AA-CHPs were prescribed for treatment of no more than seven days and the most common dosage frequency (82.7%) was three times a day. Furthermore, our investigation of the 2004 database found an alarming number of cases of CHPs containing AA herbs (*Tianxianteng *or *Madouling*) prescribed after the ban was announced on 4 November 2003. We found a total of 68 records involving the prescription of these herbs to 25 patients by 19 Chinese medicine practitioners (in 19 clinics). Therefore, our estimate was that about 2,760 patients (= 25*23,000,000* 96.16%/200,000) were prescribed with the prohibited AA-CHPs at least once during the study period.

## Discussion

This study demonstrated that more than one-third (39.3%) of the population in Taiwan were prescribed with AA-CHPs during the study period and that the cumulated doses of AA-CHPs for each patient may have exceeded 100 g (Table 3). Exposure to *Xixin *and *Mutong *was the most extensive. Therefore, it is necessary to monitor the use of CHPs. Special attention should be drawn to prescriptions for patients suffering from respiratory and/or musculoskeletal diseases and to the herbal formulae with AA herbs (Table 4).

There are a few major limitations to this study. Firstly, the study was based upon the NHI reimbursement data. Specific information is not available for causal studies or inference. Secondly, different pharmaceutical companies may obtain their herbs from different sources which may have different degrees of AA herb adulterations. The estimation of cumulated AA doses may be inaccurate. Thirdly, this study did cover the consumption of medicinal herbs purchased directly from the market. Therefore our estimate does not represent all consumption of AA herbs in Taiwan.

## Conclusion

This study showed a prescription profile of AA-CHPs in Taiwan between 1997 and 2003 based on the NHI reimbursement data, including an estimate of the total amount of AA herbs consumed and the target population requiring continuous monitoring. Moreover, this study revealed the NHI prescription of some banned AA-CHPs.

## Abbreviations

AA: aristolochic acid; CHPs: Chinese herbal products; AA-CHPs: CHPs containing AA; NHI: National Health Insurance; CCMP: Committee on Chinese Medicine and Pharmacy.

## Competing interests

The authors declare that they have no competing interests.

## Authors' contributions

SCH conducted the study design, data management, statistical analysis, preparation and revision of the manuscript. IHL contributed to the study design and coordinated the study. WLT and CHL assisted in literature survey and data interpretation. JDW conceived, designed, coordinated the study and helped draft the manuscript. All authors read and approved the final manuscript.

## References

[B1] Vanherweghem JL, Depierreux M, Tielemans C, Abramowicz D, Dratwa M, Jadoul M, Richard C, Vandervelde D, Verbeelen D, Vanhaelen-Fastre R (1993). Rapidly progressive interstitial renal fibrosis in young women: association with slimming regimen including Chinese herbs. Lancet.

[B2] Vanhaelen M, Vanhaelen-Fastre R, But P, Vanherweghem JL (1994). Identification of aristolochic acid in Chinese herbs. Lancet.

[B3] Lord GM, Cook T, Arlt VM, Schmeiser HH, Williams G, Pusey CD (2001). Urothelial malignant disease and Chinese herbal nephropathy. Lancet.

[B4] Krumme B, Endmeir R, Vanhaelen M, Walb D (2001). Reversible Fanconi syndrome after ingestion of a Chinese herbal 'remedy' containing aristolochic acid. Nephrol Dial Transplant.

[B5] Pena JM, Borras M, Ramos J, Montoliu J (1996). Rapidly progressive interstitial renal fibrosis due to a chronic intake of a herb (Aristolochia pistolochia) infusion. Nephrol Dial Transplant.

[B6] Stengel B, Jones E (1998). End-stage renal insufficiency associated with Chinese herbal consumption in France. Nephrologie.

[B7] Tanaka A, Nishida R, Yoshida T, Koshikawa M, Goto M, Kuwahara T (2001). Outbreak of Chinese herb nephropathy in Japan: are there any differences from Belgium?. Intern Med.

[B8] Chen W, Chen Y, Li A (2001). The clinical and pathological manifestations of aristolochic acid nephropathy – the report of 58 cases. Zhonghua Yixue Zazhi.

[B9] Chang CH, Wang YM, Yang AH, Chiang SS (2001). Rapidly progressive interstitial renal fibrosis associated with Chinese herbal medications. Am J Nephrol.

[B10] Chen L, Mei N, Yao L, Chen T (2006). Mutations induced by carcinogenic doses of aristolochic acid in kidney of Big Blue transgenic rats. Toxicol Lett.

[B11] Cosyns JP, Dehoux JP, Guiot Y, Goebbels RM, Robert A, Bernard AM, van Ypersele de Strihou C (2001). Chronic aristolochic acid toxicity in rabbits: a model of Chinese herbs nephropathy?. Kidney Int.

[B12] Cui M, Liu ZH, Qiu Q, Li H, Li LS (2005). Tumour induction in rats following exposure to short-term high dose aristolochic acid I. Mutagenesis.

[B13] Arlt VM, Stiborova M, Schmeiser HH (2002). Aristolochic acid as a probable human cancer hazard in herbal remedies: a review. Mutagenesis.

[B14] Cosyns JP (2003). Aristolochic acid and 'Chinese herbs nephropathy': a review of the evidence to date. Drug Saf.

[B15] Cosyns JP (2002). Human and experimental features of aristolochic acid nephropathy (AAN; Formally Chinese herbs nephropathy-CHN): are they relevant to Balkan endemic nephropath8 (BEN). Medicine and Biology.

[B16] International Agency for Research on Cancer Monographs on the evaluation of carcinogenic risks to humans – Complete list of agents evaluated and their classification.

[B17] Schwetz BA (2001). From the Food and Drug Administration. JAMA.

[B18] Cheung TP, Xue C, Leung K, Chan K, Li CG (2006). Aristolochic acids detected in some raw Chinese medicinal herbs and manufactured herbal products – a consequence of inappropriate nomenclature and imprecise labelling?. Clin Toxicol (Phila).

[B19] Kessler DA (2000). Cancer and herbs. N Engl J Med.

[B20] Committee on Chinese Medicine and Pharmacy, Department of Health Executive Yuan, Taiwan Regulations on aristolochic acid-contained Chinese herbal medicine products.

[B21] Chuang MS, Hsu YH, Chang HC, Lin JH, Liao CH (2002). Studies on adulteration and misusage of marketed akebiae caulis. Ann Rept NLFD Taiwan ROC.

[B22] Deng JS (2002). Quality evaluation of Fang-Ji and analysis of marker constituents. PhD thesis.

[B23] Hsu YH, Tseng HH, Wen KC (1997). Determination of aristolochic acid in fangchi radix. Ann Rept NLFD Taiwan ROC.

[B24] Tung CF, Ho YL, Tsai HY, Chang YH (1999). Studies on the commonly misused and adulterated Chinese crude drug species in Taiwan. Chin Med Coll J.

[B25] Ohno T, Mikami E, Matsumoto H, Kawaguchi N (2006). Identification tests of aristolochic acid in crude drugs by reversed-phase TLC/scanning densitometry. J Health Sci.

[B26] Ioset JR, Raoelison GE, Hostettmann K (2003). Detection of aristolochic acid in Chinese phytomedicines and dietary supplements used as slimming regimens. Food Chem Toxicol.

[B27] Jou J-H, Li C-Y, Schelonka EP, Lin C-H, Wu T-S (2004). Analysis of the analogue of aristolochic acid and aristolactam in the plant of aristolochia genus by HPLC. J Food Drug Anal.

[B28] Schaneberg BT, Khan IA (2004). Analysis of products suspected of containing Aristolochia or Asarum species. J Ethnopharmacol.

[B29] Hashimoto K, Higuchi M, Makino B, Sakakibara I, Kubo M, Komatsu Y, Maruno M, Okada M (1999). Quantitative analysis of aristolochic acids, toxic compounds, contained in some medicinal plants. J Ethnopharmacol.

[B30] Jong TT, Lee MR, Hsiao SS, Hsai JL, Wu TS, Chiang ST, Cai SQ (2003). Analysis of aristolochic acid in nine sources of Xixin, a traditional Chinese medicine, by liquid chromatography/atmospheric pressure chemical ionization/tandem mass spectrometry. J Pharm Biomed Anal.

[B31] U.S. Food & Drug Administration, Center for Food Safety & Applied Nutrition Dietary Supplements Aristolochic Acid.

[B32] Drew AK, Whyte IM, Bensoussan A, Dawson AH, Zhu X, Myers SP (2002). Chinese herbal medicine toxicology database: monograph on Herba Asari, "xi xin". J Toxicol Clin Toxicol.

[B33] WHO (2000). General Guidelines for Methodologies on Research and Evaluation of Traditional Medicine. Document WHO/EDM/TRM/20001.

[B34] Molony D (1998). The American Association of Oriental Medicine's Complete Guide to Chinese Herbal Medicine.

[B35] Editors Huangdi Neijing-Suwen. Document CM017.

[B36] WHO (2002). WHO Traditional medicine strategy 2002–2005. Document WHO/EDM/TRM/20021.

[B37] Guh JY, Chen HC, Tsai JF, Chuang LY (2007). Herbal therapy is associated with the risk of CKD in adults not using analgesics in Taiwan. Am J Kidney Dis.

[B38] Hsieh SC, Lai JN, Lee CF, Tseng WL, Hu FC, Wang JD (2008). The prescribing of Chinese herbal products in Taiwan: a cross-sectional analysis of the national health insurance reimbursement database. Pharmacoepidemiol Drug Saf.

[B39] Kung YY, Chen YC, Hwang SJ, Chen TJ, Chen FP (2006). The prescriptions frequencies and patterns of Chinese herbal medicine for allergic rhinitis in Taiwan. Allergy.

[B40] Committee on Chinese Medicine and Pharmacy, Department of Health Executive Yuan, Taiwan List of 100 unified formulas.

[B41] Bureau of National Health Insurance, Taiwan The National Health Insurance Statistics – Beneficiaries profile.

[B42] Knuth DE (1997). Art of Computer Programming, Seminumerical Algorithms.

[B43] Park SK, Miller KW (1988). Random Number Generators: Good Ones are Hard to Find. CACM.

[B44] Bureau of National Health Insurance, Taiwan International publications regarding the use of national health insurance database Taiwan.

